# Supplementary material for: Altered microRNA Transcriptome in Cultured Human Liver Cells upon Infection with Ebola Virus
Source: Int J Mol Sci. 2021 Apr 6;22(7):3792. doi: 10.3390/ijms22073792 (PMC8038836; doi:10.3390/ijms22073792)
Supplement: Supplementary file 1 [file ijms-22-03792-s001.zip › Supplementary_File/D_ KEGG_Analysis_Results/16-30nt_pathway_Mayinga-24h-Huh7_vs_Control-24h-Huh7_up.mature_mirna_targets/hsa_EnrichmentScoreDotPlot.pdf]

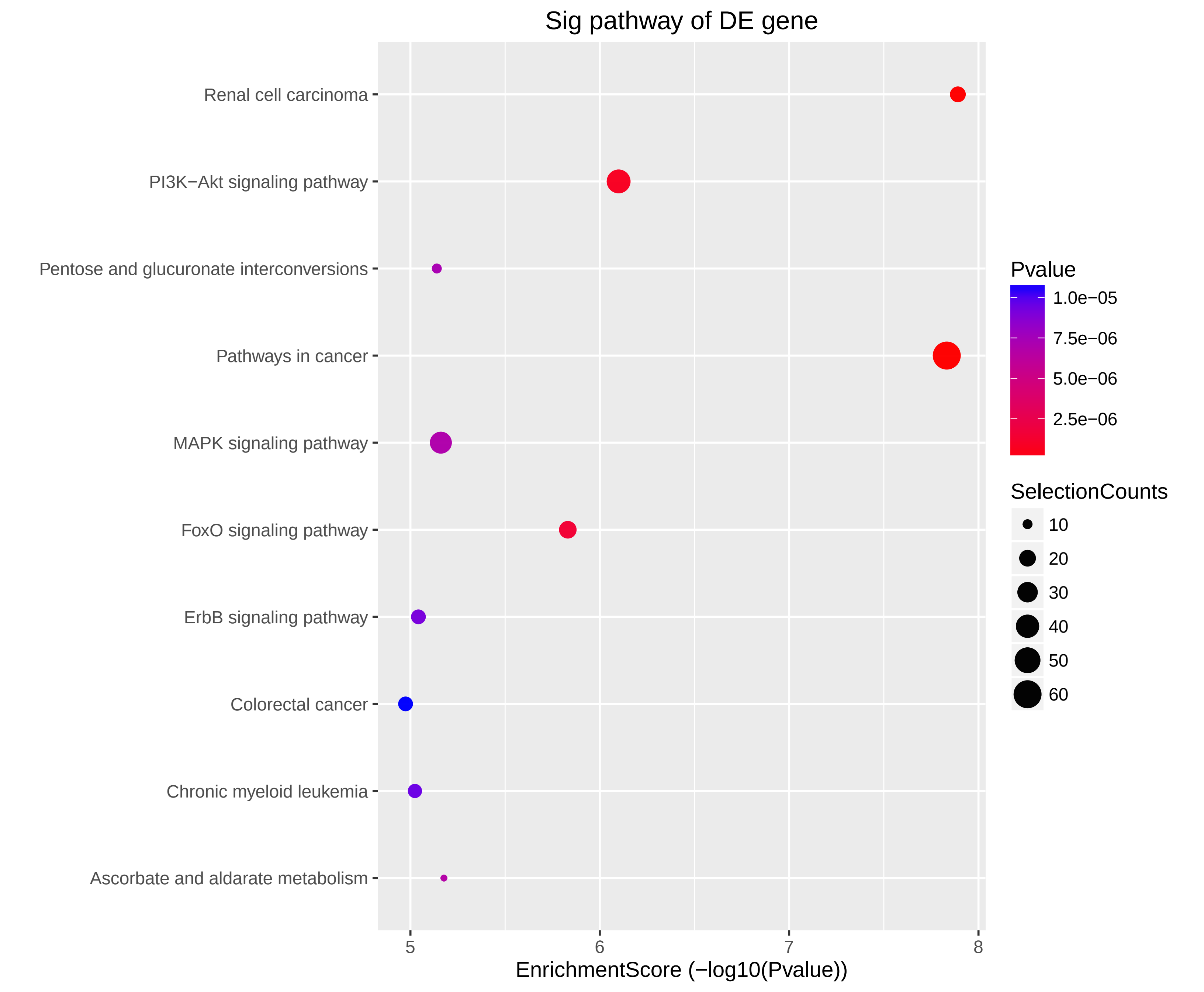

Sig pathway of DE gene
Renal cell carcinoma
PI3K−Akt signaling pathway
Pentose and glucuronate interconversions
Pathways in cancer
MAPK signaling pathway
FoxO signaling pathway
ErbB signaling pathway
Colorectal cancer
Chronic myeloid leukemia
Ascorbate and aldarate metabolism
5
6
7
8
EnrichmentScore (−log10(Pvalue))
Pvalue
1.0e−05
7.5e−06
5.0e−06
2.5e−06
SelectionCounts
10
20
30
40
50
60
